# Differential regulation of lung homeostasis and silicosis by the TAM receptors MerTk and Axl

**DOI:** 10.3389/fimmu.2024.1380628

**Published:** 2024-05-07

**Authors:** Kamila Guimarães-Pinto, Monique Leandro, Antonia Corrêa, Ester P. Maia, Leticia Rodrigues, André Luiz Amorim da Costa, Jesuino Rafael Machado Ferreira, Estefannia Claudio-Etienne, Ulrich Siebenlist, Jianping He, Thaís da Silva Rigoni, Tatiana Paula Teixeira Ferreira, Yago Amigo Pinho Jannini-Sa, Herbert Leonel Matos-Guedes, Ana Caroline Costa-da-Silva, Marcela Freitas Lopes, Patricia Machado Rodrigues Silva, Brian Lee Kelsall, Alessandra Almeida Filardy

**Affiliations:** ^1^ Institute of Microbiology, Center for Health Science, Federal University of Rio de Janeiro, Rio de Janeiro, RJ, Brazil; ^2^ Institute of Biophysics Carlos Chagas Filho, Center for Health Science, Federal University of Rio de Janeiro, Rio de Janeiro, RJ, Brazil; ^3^ Laboratory of Allergic Diseases, National Institute of Allergy and Infectious Diseases, National Institutes of Health (NIH), Maryland, MD, United States; ^4^ Mucosal Immunobiology Section, Laboratory of Molecular Immunology, National Institute of Allergy and Infectious Diseases, National Institutes of Health (NIH), Maryland, MD, United States; ^5^ Laboratório de Inflamação, Instituto Oswaldo Cruz, Fundação Oswaldo Cruz, Rio de Janeiro, Brazil; ^6^ Laboratório de Imunobiotecnologia, Instituto de Microbiologia Paulo de Góes, Universidade Federal do Rio de Janeiro, Rio de Janeiro, Brazil; ^7^ Laboratório de Imunologia Clínica, Instituto Oswaldo Cruz, Fundação Oswaldo Cruz, Rio de Janeiro, Brazil; ^8^ Oral Immunobiology Unit, National Institute of Dental and Craniofacial Research, National Institutes of Health (NIH), Maryland, MD, United States

**Keywords:** alveolar macrophage, efferocytosis, immunoregulation, silicosis, airways homeostasis

## Abstract

**Introduction:**

TAM receptor-mediated efferocytosis plays an important function in immune regulation and may contribute to antigen tolerance in the lungs, a site with continuous cellular turnover and generation of apoptotic cells. Some studies have identified failures in efferocytosis as a common driver of inflammation and tissue destruction in lung diseases. Our study is the first to characterize the *in vivo* function of the TAM receptors, Axl and MerTk, in the innate immune cell compartment, cytokine and chemokine production, as well as the alveolar macrophage (AM) phenotype in different settings in the airways and lung parenchyma.

**Methods:**

We employed MerTk and Axl defective mice to induce acute silicosis by a single exposure to crystalline silica particles (20 mg/50 μL). Although both mRNA levels of Axl and MerTk receptors were constitutively expressed by lung cells and isolated AMs, we found that MerTk was critical for maintaining lung homeostasis, whereas Axl played a role in the regulation of silica-induced inflammation. Our findings imply that MerTk and Axl differently modulated inflammatory tone via AM and neutrophil recruitment, phenotype and function by flow cytometry, and TGF-β and CXCL1 protein levels, respectively. Finally, Axl expression was upregulated in both MerTk^-/-^ and WT AMs, confirming its importance during inflammation.

**Conclusion:**

This study provides strong evidence that MerTk and Axl are specialized to orchestrate apoptotic cell clearance across different circumstances and may have important implications for the understanding of pulmonary inflammatory disorders as well as for the development of new approaches to therapy.

## Introduction

The phagocytosis of apoptotic cells, termed efferocytosis, is critical for maintaining tissue homeostasis, inflammation, and autoimmune responses, particularly in environments with high cell turnover and apoptosis, such as the lungs (1–3). Indeed, defects in efferocytosis have been identified as a common cause of inflammation and tissue destruction in inflammatory lung diseases (4–6). Alveolar macrophages (AMs) are the most abundant professional phagocytes in the alveolar space and express multiple apoptotic cell recognition receptors, emphasizing their importance as a key efferocytic cell in this microenvironment (2, 5, 7). The Tyro3/Axl/MerTk (TAM) family of receptor tyrosine kinases (RTKs) recognizes phosphatidylserine (PtdSer) on apoptotic cells via the bridging molecules Growth-arrest-specific 6 (Gas6) and protein S (PROS1). Once activated, TAM receptors mediate apoptotic cell clearance and also inhibit inflammatory responses via a negative feedback involving activation of suppressor of cytokine signaling 1 (SOCS1) and SOCS3 on phagocytes (8–10). The suppressive role of TAM receptor signaling emphasizes their importance in immune regulation, contributing to microbiota tolerance in sites with a high number of apoptotic cells, such as the lung mucosae. Indeed, our prior studies have identified neutrophil efferocytosis followed by a challenge with bacterial LPS as important for driving the differentiation of regulatory macrophages (11). Although Axl and/or MerTK are preferentially expressed in the immune system cells (12), some evidence suggests that TAM receptors are selectively expressed in macrophage populations. MerTk is expressed by all mature tissue macrophage populations during homeostasis, whereas Axl is expressed by airways macrophages, spleen red pulp macrophages, and Kupffer cells (13–16). Furthermore, some *in vitro* studies have shown that Axl and MerTk play distinct functions in health and disease, and that they are dynamically regulated on macrophages by pro- and anti-inflammatory stimuli (13, 14). Despite these studies, the impact of TAM receptor signaling in regulating innate immune cell populations, cytokine and chemokine production as well as AM phenotype in both *in vivo* steady state and inflammatory settings in the lungs has not been addressed yet.

Here, we showed that lung cells and isolated AMs express both the Axl and MerTk receptors constitutively, and that MerTk is critical for maintaining lung homeostasis by regulating neutrophil and AM recruitment, inflammatory AM phenotype and function, and maintaining high levels of TGF-β and IL-10 in the airways. Additionally, we demonstrated that the absence of Axl impaired the control of silica-induced inflammation, leading to an increase in inflammatory cells, pro-fibrotic TGF-β and CXCL1, as well as apoptotic and necrotic cell debris, and a decrease in the number of non-inflammatory AMs. Finally, we showed that both MerTk^-/-^ and WT AMs have elevated Axl mRNA, highlighting the significance of this receptor in efferocytosis and inflammatory modulation. This study provides evidence that MerTk and Axl are specialized to orchestrate apoptotic cell clearance in different settings, where they play constitutive and significant roles in lung immunological homeostasis and silicosis disease resolution, respectively.

## Methods

### Reagents and antibodies

Annexin V and 7-AAD were obtained from BD (Franklin Lakes, NJ) and Live/Dead Aqua from Life Technologies Corporation (Eugene, OR). Antibodies used in the study were the following: Fc block/anti-CD16/32 (2.4G2) (BD) and directly conjugated monoclonal antibodies (mAb/clone): CD45.2/104 (Invitrogen, Grand Island, NY), SiglecF/E50-2440, Ly6G/1A8, Ly6C/HK1.4, Axl/175128 (all from BD), F4/80/BM8, MHCII/M5-114.15.2, CD206/C068C2, MerTk/DS5MMER (all from Biolegend, San Diego, CA), CD11c/N418 and CD11b/M1-70 (both from eBioscience, San Diego, CA).

### Mice

Male C57BL/6 wild-type (WT), Axl^-/-^, and MerTk^-/-^ mice (8-10 weeks old) were kindly donated by Dr. Brian Kelsall, NIAID, NIH, Bethesda, MD. All mice were bred and housed in a pathogen-free animal facility of the Universidade Federal do Rio de Janeiro, under a light/dark cycle in individually ventilated cages with freely available food and water. All experimental procedures were revised and approved by the Ethics Committee for Use of Animals of the Universidade Federal do Rio de Janeiro, under protocol No. 041/17.

### Silicosis induction

Mice were intratracheally (i.t.) instilled with either 20 mg/50 μL of crystalline silica (SiO_2_, particle size 0.5-10 μm; Sigma-Aldrich, St Louis, MO) or sterile PBS (17–19). Animals were randomly divided into control (PBS-) and silicosis (SIL-) groups (3–5 mice per group). Mice survival and body weight measurements were analyzed every three days for fifteen days post-silica or PBS instillation.

### Non-invasive pulmonary mechanics measurement

Seven days following silica or PBS instillation, lung function was measured without provocation (baseline) by using barometric whole-body plethysmography (Buxco Research System, Wilmington, NC, USA) as previously described (20). Briefly, mice were placed in a whole-body chamber and allowed to stabilize for five minutes. The increased airflow resistance was measured as enhanced pause (Penh) and was assessed during a five-minute data collection period for each conscious animal during spontaneous breathing.

### Histological analysis

Left superior lobes (five-micron tissue sections) were stained with Hematoxylin and Eosin (H&E) or Picrosirius Red. For the quantification of lung parenchyma inflammatory areas, the parenchyma was submitted to automated analysis to detect and quantify the total lung left superior lobe area, which was turned into a Region of Interest (ROI). The software Smart Segmentation tool (Machine Learning) detected high-density areas (cell inflammatory infiltrates) and quantified the total area in each lobe. The total percentage of space occupied by cell inflammatory infiltrates was calculated as the equation: total cell inflammatory infiltrate area x 100/total lung area. Additional details are provided in the online **Supplementary Methods**.

### Bronchoalveolar lavage and isolation of lung parenchyma cells

Lavage of the upper airways was performed with 1 mL of sterile PBS. Cell-free supernatants from the first bronchoalveolar lavage fluid (BALF) containing airways cells were stored at -80°C for further analyses. Alternatively, lungs were perfused, removed from the thoracic cavity, and digested with collagenase VIII (100 units/mL) (Sigma-Aldrich) for 1 hour. Single-cell suspensions from lung parenchyma (lung cells) or from BALF (airway cells) were counted for further analyses. Additional details are provided in the online **Supplementary Methods**.

### Isolation of macrophages from the colon and peritoneal cavity and differentiation of macrophages from bone marrow precursors

Colonic macrophage isolation (cMPs) was prepared as described previously (21). Resident pMPs were obtained by washing the peritoneal cavity of mice with cold RPMI 1640 (Gibco). Bone marrow-derived macrophages (BMDMs) were generated by flushing femurs and tibias with PBS, and precursor cells were cultured in complete RPMI 1640 media (Gibco) supplemented with 30% L929 cell-conditioned medium. Additional details are provided in the online **Supplementary Methods**.

### RNA isolation and real-time reverse transcriptase–PCR

Total RNA was extracted from lung tissue or isolated cells using RNeasy Mini Kit (Qiagen, Valencia, CA) and reverse transcribed into cDNA using qScript cDNA Supermix (Quanta Biosciences, Beverly, MA). Real-time PCR reactions were performed using FAM-labeled gene-specific probes and primers (all from Applied Biosystems, MA, USA) on a 7900HT Sequence Detection System with TaqMan primer sets for GAS6, TYRO3, AXL, MERTK, CXCL1, CXCL2, TNF, IL-6 and GAPDH transcript levels as described in the online **Supplementary Methods**.

### Protein and cytokines quantification

Total protein concentration and cytokines concentrations were measured in the BALF by using the Micro BCA Protein Assay Kit (Thermo Scientific, MA, USA) and conventional double-sandwich enzyme-linked immunosorbent assay (ELISA) kits from eBioscience (active TGF-β), R&D (Mineapolis, MN) (IL-10) or Peprotech (Cranbury, NJ) (CXCL1), respectively. Plates were immediately read by spectrophotometer (SpectraMax M5, Molecular Devices, San Jose, CA, USA) at 562 nm (total protein) or at 450 nm (cytokines). Additional details are provided in the online **Supplementary Methods**.

### Nitric oxide assay

Production of nitric oxide (NO) was assayed indirectly by the quantification of nitrites accumulated in the first bronchoalveolar lavage by using the Griess colorimetric method.

### Statistical analyses

Data were analyzed by using GraphPad Prism software (v. 7.0). Experiments were done at least in triplicate, and the points were pooled together for statistical analysis. Statistical analysis was presented as the mean with SD. All our datasets had a normal distribution as shown by the Shapiro-Wilk normality test. Differences between two experimental groups were analyzed using unpaired Student’s two-tailed *t*-test. Differences between three or more groups were analyzed using a one-way analysis of variance (ANOVA) with a Tukey’s post-test. Statistical significance was considered as p<0,05 and reported in the figures.

## Results

### Axl and MerTk receptors are highly expressed in the lungs and by alveolar macrophages during homeostasis

**Figure 1:**
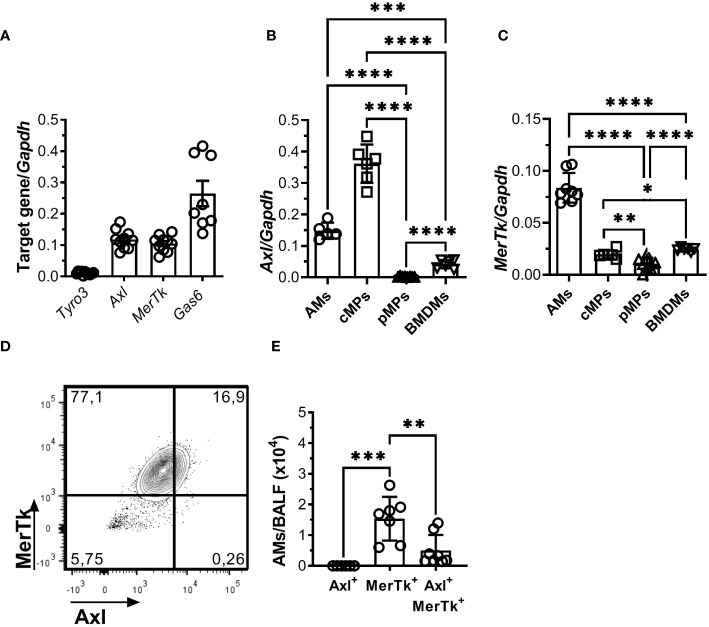
The mucosal microenvironment regulates TAM receptors’ expression pattern during homeostasis. **(A)** TAM receptors and their ligand, Gas6, mRNA expression in isolated total lung cells from WT mice. Data are shown as means ± SD. **(B, C)** Axl and MerTk mRNA expression in different macrophage subsets (AMs, cMPs, pMPs, and BMDMs) from WT mice by RT-qPCR. **(D)** Representative FACS plot of Axl^+^, MerTk^+^, and Axl^+^MerTk^+^ cells gated on AMs (SiglecF^+^CD11c^+^) from WT BALFs. **(E)** Absolute number of Axl^+^, MerTk^+^, and Axl^+^MerTk^+^ of AMs. **(A-D)** Data were obtained from three independent experiments with similar results (n ≥ 7 per group). **(E)** Data shown were combined from three independent experiments with similar results (n ≥ 6 per group). Data are shown as means ± SD. Statistical differences were analyzed by unpaired *t-*tests **(B, C)** or one-way ANOVA with Tukey’s multiple comparison test **(E)** and depicted as *p<0.05, **p<0.01, ***p<0.001, ****p<0.0001. AMs, Alveolar macrophages; cMPs, colonic macrophages; pMPs, peritoneal macrophages; BMDM: bone marrow-derived macrophages.

To evaluate the role of TAM receptors in lung homeostasis, we first profiled the mRNA expression of TAM receptors and their ligand Gas6 in the lungs of WT mice. During homeostasis, we found high levels of Axl, MerTk, and Gas6 mRNA, but negligible levels of Tyro3 mRNA in the lung cells (**Figure 1A**). We neither find increased compensatory transcription of Axl mRNA in the lung cells of MerTk-deficient mice nor MerTk mRNA in the lung cells of Axl-deficient mice (Data not shown). Next, the expression of TAM receptors and Gas6 was investigated in purified AMs, and their mRNA profiles were compared with those of macrophages from another mucosal site, colonic macrophages (cMPs), as well as with those from non-mucosal sites such as peritoneal macrophages (pMPs) and bone marrow-derived macrophages (BMDMs). A distinct cell-type-specific pattern of TAM receptor expression was observed: higher levels of Axl mRNA were preferentially expressed by the mucosal macrophages AMs and cMPs compared to pMPs and BMDMs, whereas MerTk mRNA expression was higher on AMs, despite being evenly distributed across all macrophage populations studied (**Figures 1B, C**). Furthermore, while AMs express both Axl and MerTk receptor mRNA, we found that higher number of AMs expressed only MerTk during steady state (**Figures 1D, E**). We also found that when compared to cMPs, AMs had negligible levels of Tyro 3 and low levels of Gas6 (Data not shown). These findings suggest that the mucosal milieu contains unique factors that regulate TAM receptor expression in macrophages, emphasizing their important role in lung homeostasis.

### MerTk has a unique role in regulating cytokine production and inflammatory cell recruitment to the airways during steady state

**Figure 2:**
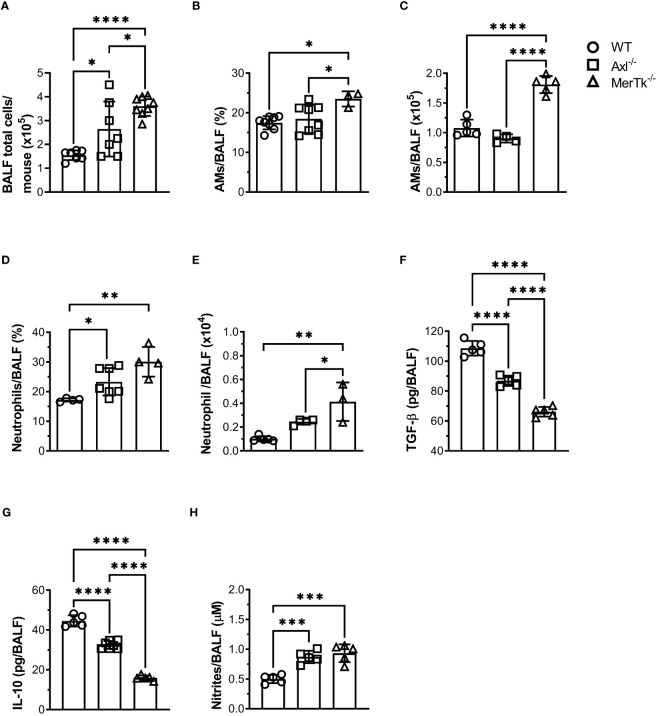
MerTk receptor deficiency causes airway homeostasis imbalance. **(A)** Total BALF cell numbers from WT, Axl^-/-^, and MerTk^-/-^ mice. Data are shown as means ± SD. **(B, C)** Frequencies and absolute numbers of AMs (SiglecF^+^CD11c^+^) of total live cells gate from WT, Axl^-/-^, and MerTk^-/-^ mice BALFs determined by flow cytometry. **(D, E)** Frequencies and absolute numbers of neutrophils (SiglecF^-^CD11c^-^Ly6G^+^) within CD11b^+^ gate from WT, Axl^-/-^, and MerTk^-/-^ mice BALFs analyzed by flow cytometry. **(F)** Levels of active TGF-β, **(G)** IL-10, and **(H)** nitrites in BAL fluids from WT, Axl^-/-^, and MerTk^-/-^ mice, measured by ELISA or Griess Assay, respectively. **(A, F-H)** Data were obtained from four independent experiments with similar results (n ≥ 5 per group). **(B-E)** Data were obtained from three independent experiments with similar results (n ≥ 3 per group). Data are shown as means ± SD. Statistical differences were analyzed by one-way ANOVA with Tukey’s multiple comparison test **(A-H)** and depicted as *p<0.05, **p<0.01, ***p<0.001, ****p<0.0001. AMs, Alveolar macrophages; BALFs, Bronchoalveolar lavage fluid.

We next investigated how TAM receptors affect the number and proportion of airway cells, as well as the cytokine profile in BALFs. Higher numbers of airway cells were observed in the BALFs of both Axl and MerTk deficient compared to WT mice, with even greater numbers in MerTk compared to Axl deficient mice (**Figure 2A**). Characterization of BALFs cell populations by flow cytometry profiling showed that MerTk-deficient mice had higher percentages and absolute numbers of AMs than WT or Axl-deficient mice (**Figures 2B, C**). Furthermore, we found higher percentages and absolute numbers of neutrophils in both Axl and MerTk-deficient mice compared to WT mice (**Figures 2D, E**). Higher levels of nitrites and lower levels of the regulatory cytokines TGF-β and IL-10 in the BALFs of both Axl and MerTk-deficient mice compared to WT mice confirmed this inflammatory profile (**Figures 2F-H**). Interestingly, in parallel with higher recruitment of neutrophils, we found even lower levels of TGF-β and IL-10 in the BALFs of MerTk compared to Axl-deficient mice (**Figures 2F, G**). These findings support the role of efferocytosis in regulating proinflammatory and regulatory molecules. Taken together, our data suggest that the absence of Axl or MerTk receptors causes an imbalance between resident cell populations and inflammatory cell recruitment. This shift may be attributed to the reduced production of regulatory molecules and/or accumulation of dead cells in the airway space during steady state, with MerTk exerting a more prominent role controlling airway homeostasis.

### MerTk regulates cytokine expression and inflammatory cell recruitment to the lung parenchyma during homeostasis

**Figure 3:**
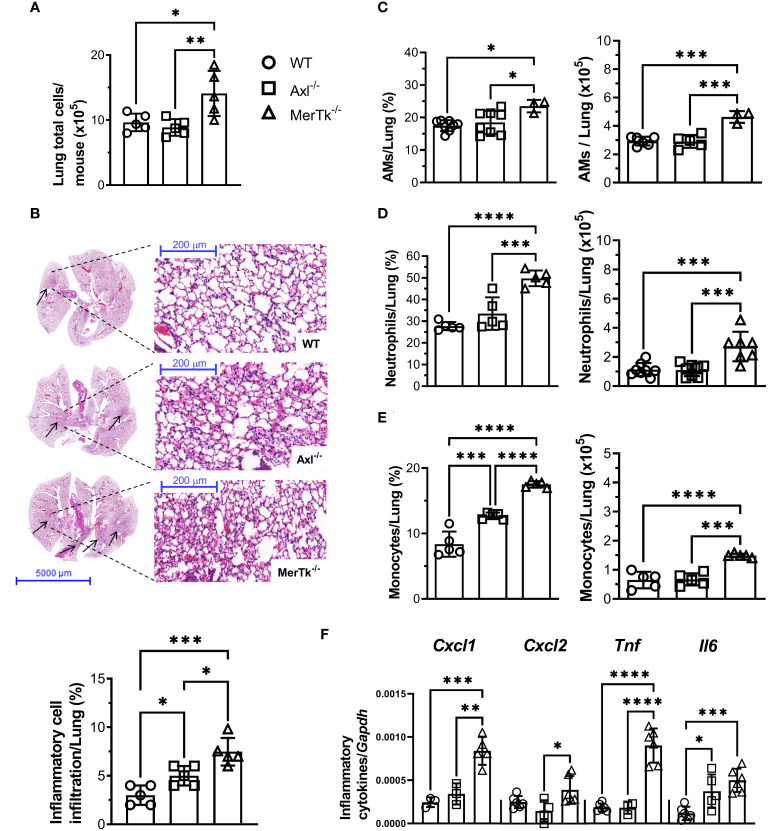
MerTk receptor deficiency causes lung parenchyma homeostasis imbalance. **(A)** Total lung cell numbers from WT, Axl^-/-^, and MerTk^-/-^ mice. **(B)** Representative lung histology images and percentage of lung area occupied by inflammatory cell recruitment based on H&E staining in WT, Axl^-/-^, and MerTk^-/-^ mice. Frequencies and absolute numbers of **(C)** AMs (SiglecF^+^CD11c^+^) of total live cells gate, **(D)** neutrophils (SiglecF^-^CD11c^-^Ly6G^+^), and **(E)** monocytes (SiglecF^-^CD11c^-^Ly6C^+^) within CD11b^+^ gate from total lung tissue of WT, Axl^-/-^, and MerTk^-/-^ mice acquired by flow cytometry. **(F)** Total lung cells mRNA expression of CXCL1, CXCL2, TNF-α, and IL-6 from WT, Axl^-/-^, and MerTk^-/-^ mice analyzed by RT-qPCR. **(A, B)** Data were obtained from three independent experiments with similar results (n = 5 per group). **(C-F)** Data were obtained from four independent experiments with similar results (n ≥ 3 per group). Data are shown as means ± SD. Statistical differences were analyzed by one-way ANOVA with Tukey’s multiple comparison test **(A-F)** and depicted as *p<0.05, **p<0.01, ***p<0.001, ****p<0.0001. AMs, Alveolar macrophages.

To verify whether the lung parenchyma is also altered in Axl and MerTk deficient mice in the steady state condition, we investigated lung structure and cellular infiltration during homeostasis. We first observed that MerTk-deficient mice had more total lung parenchyma cells than WT or Axl-deficient mice (**Figure 3A**). To assess the lung parenchyma structure, we performed Hematoxylin-Eosin staining to identify and quantify the number of lung areas matching inflammatory or cell recruitment areas. Analysis of total left superior lung lobe showed increased inflammatory foci in MerTk-deficient mice than in WT and Axl-deficient mice (**Figure 3B**, upper panels). Next, we quantified that MerTk-deficient mice have more inflammatory infiltrates in the lung parenchyma than WT and Axl-deficient mice (**Figure 3B**, lower panel). We further characterized the cell infiltration by flow cytometry and found that MerTk-deficient mice had a higher percentage and absolute number of AMs (**Figure 3C**), neutrophils (**Figure 3D**), and monocytes (**Figure 3E**) in the lung parenchyma than WT or Axl-deficient mice. In addition, higher levels of CXCL1 and CXCL2 chemokines transcripts, which play important roles in recruiting neutrophils and monocytes, respectively, were detected in the lung parenchyma of MerTk-deficient mice compared to WT or Axl-deficient mice, along with TNF and IL-6, cytokines implicated in tissue damage (**Figure 3F**).

### MerTk controls alveolar macrophage phenotype and lung parenchyma homeostasis

**Figure 4:**
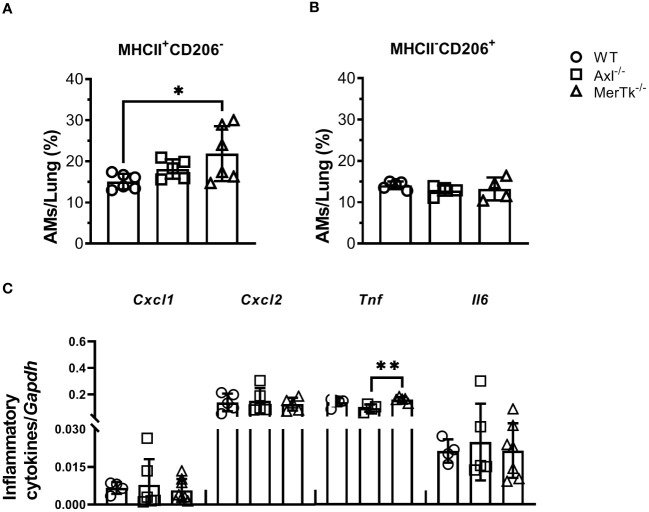
Expression of the MerTk receptor regulates AMs functional phenotype during homeostasis. **(A, B)** MHCII and CD206 expression in BALF-isolated AMs (SiglecF^+^CD11c^+^) from WT, Axl^-/-^, and MerTk^-/-^ mice. **(C)** CXCL1, CXCL2, TNF, and IL-6 mRNA expression by RT-qPCR of AMs from WT, Axl^-/-^, and MerTk^-/-^ mice. **(A-C)** Data were obtained from two independent experiments with similar results (n ≥ 4per group). Data are shown as means ± SD. Statistical differences were analyzed by one-way ANOVA with Tukey’s multiple comparison test **(A-C)** depicted as *p<0.05, **p<0.01. AMs, Alveolar macrophages.

To better understand the role of Axl and MerTk receptors in AM function within the lung milieu, we assessed the phenotype of AMs. MerTk-deficient mice had a higher frequency of M1-like MHCII^+^ AMs than Axl-deficient or WT mice, but there were no differences in the frequency of M2-like CD206^+^ AMs (**Figures 4A, B**). Furthermore, mRNA expression of CXCL1, CXCL2, TNF, and IL-6 was not increased in AMs from Axl-deficient mice and MerTk-deficient mice compared to WT mice (**Figure 4C**), suggesting that they are not the source of these inflammatory molecules found in the lung parenchyma. Overall, these findings suggest that MerTk is sufficient for limiting inflammatory cell recruitment to the lungs, preserving lung parenchyma structure and function, and maintaining the regulatory phenotype of AMs under homeostatic settings.

### Axl receptor controls lung inflammation during silicosis

**Figure 5:**
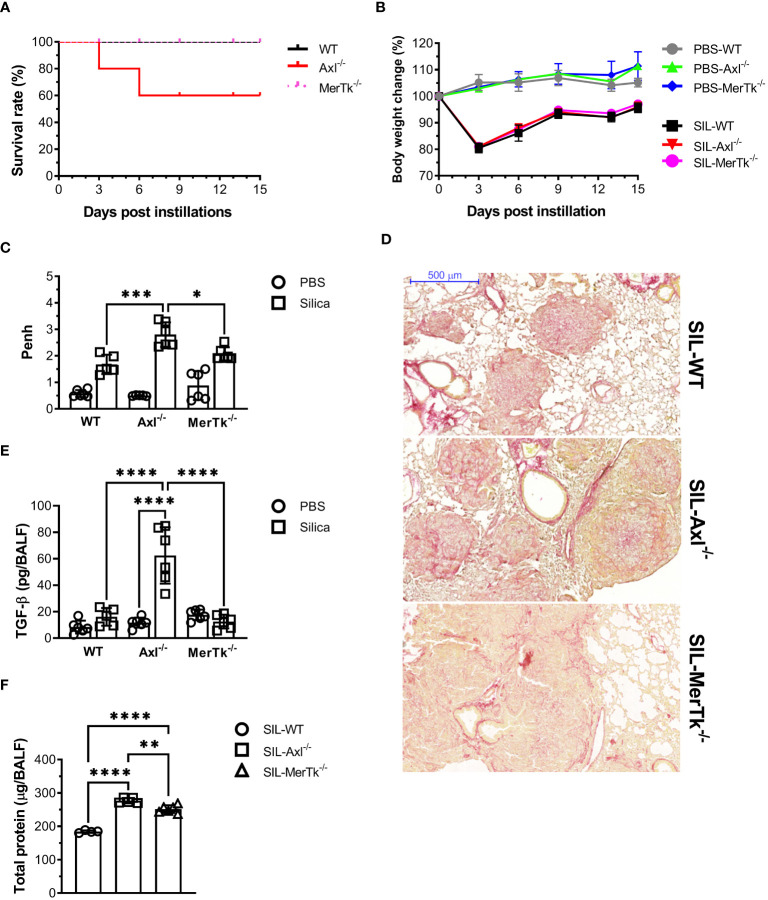
Acute induced-silica inflammation disrupts pulmonary structure and function in the airways. WT, Axl^-/-^, and MerTk^-/-^ mice were i.t. injected with 20 mg/50 µL of a sterile silica suspension or 50 µL of PBS. Mice were sacrificed 15 days after the injection. **(A)** Survival curve and **(B)** body weight change of mice over 15 days following PBS or silica i.t. instillation. **(C)** Non-invasive Penh measurements of airway resistance on day 7 following PBS or silica i.t. injection measured by whole-body plethysmography (Buxco system). **(D)** Representative images of lung lobes stained with Picro Sirius from silica WT, Axl^-/-^, and MerTk^-/-^ mice for assessment of visible collagen deposition areas. Scale bars = 500 μm. **(E)** ELISA of active TGF-β amounts and **(F)** total protein levels in BALFs from WT, Axl^-/-^, and MerTk^-/-^ mice following PBS or silica i.t. injection. **(A-F)** Data were obtained from four independent experiments with similar results (n ≥ 4per group). Data are shown as means ± SD. Statistical differences were analyzed by one-way ANOVA with Tukey’s multiple comparison test and depicted as *p<0.05, **p<0.01, ***p<0.001, ****p<0.0001. BALFs, Bronchoalveolar lavage fluid.

The role of Axl and MerTk receptors was also evaluated during lung inflammation using a murine model of acute silicosis via surgical intratracheal instillation of silica in WT (SIL-WT), Axl (SIL-Axl), and MerTk (SIL-MerTk) deficient mice and evaluated pulmonary damage in the first 15 days after instillation. Survival rate and body weight were measured every 3 days. We found a 40% decrease in survival rate only in the SIL-Axl group (**Figure 5A**), and a significant weight loss during the first three days after silica instillation, with an equal recovery of about 95% of the initial body weight in all three groups of mice by day 15 (**Figure 5B**). We also assessed the pulmonary function by whole-body plethysmograph and found that the SIL-Axl group had a higher enhanced pause response (Penh) index peak at day 7 post-silica exposure than the SIL-MerTk or SIL-WT groups (**Figure 5C**), which suggests airway resistance. Although we did not quantify collagen fibers, analysis of lung sections stained with Picro Sirius Red supports an increase in collagen content, which may be associated with lung parenchyma damage in the SIL-Axl group compared to the SIL-MerTk or SIL-WT groups (**Figure 5D**). Supporting this, we found higher levels of the pro-fibrotic cytokine TGF-β (**Figure 5E**) as well as increased lung permeability due to higher protein levels in the BALF of SIL-Axl compared to SIL-MerTk or SIL-WT (**Figure 5F**). Together, these findings suggest that a lack of Axl receptor function increases the lung susceptibility to developing severe silicosis.

### Axl receptor controls lung inflammatory cell recruitment in the airways during murine silicosis

**Figure 6:**
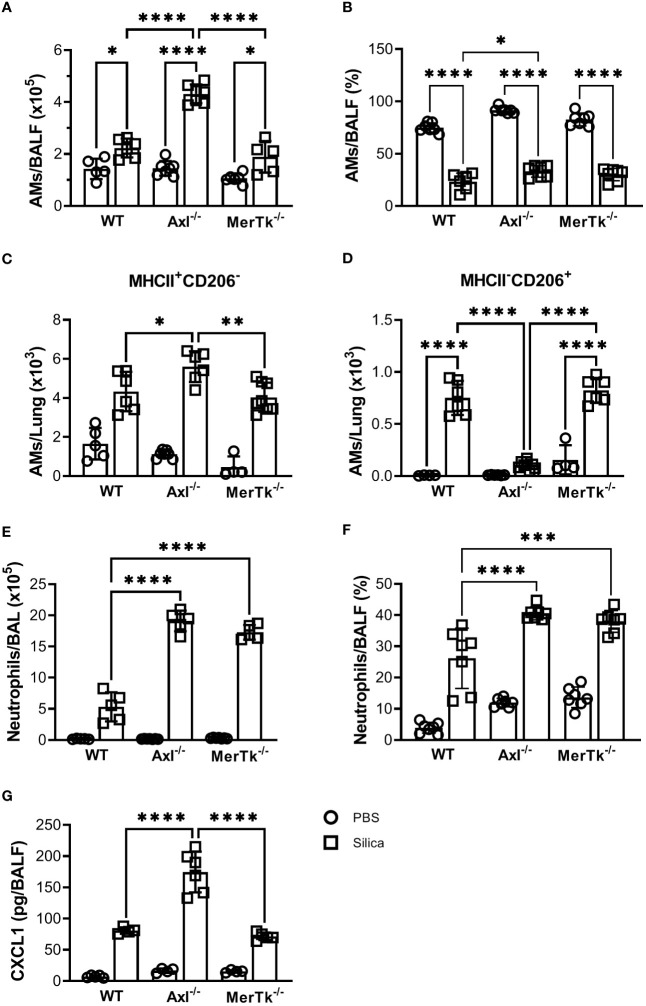
Silica-induced inflammation enhanced airway cell recruitment and is associated with AMs phenotype shift. **(A)** Absolute numbers and **(B)** frequencies of AMs (SiglecF^+^CD11c^+^) of total live cells from WT, Axl^-/-^, and MerTk^-/-^ mice BALFs determined by flow cytometry 15 days following PBS or silica i.t. injection. **(C)** Frequencies of MHCII^+^ and **(D)** CD206^+^ AMs analyzed by flow cytometry from WT, Axl^-/-^, and MerTk^-/-^ mice BALFs on day 15 following i.t. PBS or silica administration. **(E)** Absolute numbers and **(F)** frequencies of neutrophils (SiglecF^-^CD11c^-^Ly6G^+^) within CD11b^+^ gate from WT, Axl^-/-^, and MerTk^-/-^ mice BALFs determined by flow cytometry 15 days following PBS or silica i.t. injection. **(G)** ELISA of CXCL1 amounts in BAL fluids from WT, Axl^-/-^, and MerTk^-/-^ mice following PBS or silica i.t. injection. **(A, B, E, F)** Data were obtained from three independent experiments with similar results (n ≥ 5 per group). **(C, D, G)** Data were obtained from four independent experiments with similar results (n ≥ 4 per group). Data are shown as means ± SD. Statistical differences were analyzed by one-way ANOVA with Tukey’s multiple comparison test and depicted as *p<0.05, **p<0.01, ***p<0.001, ****p<0.0001. AMs, Alveolar macrophages; BALFs, Bronchoalveolar lavage fluid.

To better characterize the inflammatory cell populations recruited to the lungs during silicosis, we performed flow cytometry of the BALFs 15 days after silica exposure. SIL-Axl BALFs had a higher number of AMs than the SIL-WT group (**Figure 6A**). However, although all SIL groups exhibited a lower percentage of AMs than their PBS counterparts, the BALFs of SIL-Axl were found to contain a higher percentage of AMs than those of the SIL-WT group (**Figure 6B**). Functionally, we found a significant increase in the number of M1-like MHCII^+^CD206^-^ and a severe decrease in the number of M2-like CD206^+^MHCII^-^ AMs in the SIL-Axl group when compared to the SIL-MerTk or SIL-WT groups (**Figures 6C, D**). Furthermore, we observed a higher number and percentage of neutrophils, followed by silica instillation in the BALFs of both SIL-Axl and SIL-MerTk compared to SIL-WT group (**Figures 6E, F**). However, increased CXCL1 levels were found in the BALFs of SIL-Axl compared to SIL-MerTk or SIL-WT groups (**Figure 6G**). These data suggest that during silicosis, both Axl and MerTk regulate neutrophil recruitment, but only Axl modulates AM polarization and function.

### Axl receptor controls efferocytosis in the airways during murine silicosis

**Figure 7:**
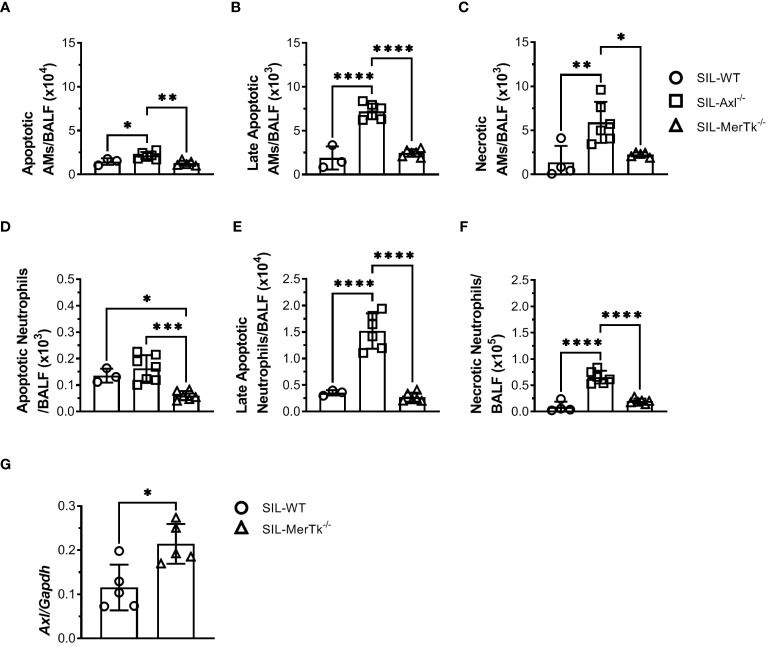
Axl receptor regulates apoptotic cell removal during acute silica inflammation. **(A-C)** Absolute numbers of apoptotic (Annexin V^+^7AAD^-^), late apoptotic (Annexin V^+^7AAD^+^), and necrotic (Annexin V^-^7AAD^+^) AMs (SiglecF^+^CD11c^+^) and **(D-F)** neutrophils (Ly6G^+^CD11b^+^) from WT, Axl^-/-^, and MerTk^-/-^ mice by flow cytometry 15 days following PBS or silica i.t. injection. **(G)** Axl mRNA expression by RT-qPCR of total lung cells from WT, and MerTk^-/-^ mice 15 days following silica i.t. injection. **(A-G)** Data were obtained from three independent experiments with similar results (n ≥ 4 per group). Data are shown as means ± SD. Statistical differences were analyzed by one-way ANOVA with Tukey’s multiple comparison test **(A-F)** or unpaired *t-*tests **(G)** and depicted as *p<0.05, **p<0.01, ***p<0.001, ****p<0.0001. AMs, Alveolar macrophages; BALFs, Bronchoalveolar lavage fluid.

Due to the important role of Axl and MerTk receptors in the clearance of apoptotic cells, as well as in the resolution of inflammation, we then looked at whether apoptotic cells were accumulated in the BALFs during silicosis. SIL-Axl BALFs had increased apoptotic (AnnexinV^+^PI^-^), late apoptotic (AnnexinV^+^PI^+^), and necrotic (AnnexinV^-^PI^+^) AMs (**Figures 7A-C**) and neutrophils (**Figures 7D-F**) in comparison to SIL-MerTk or SIL-WT groups. Finally, a significant increase in Axl expression in SIL-MerTk^-/-^ AMs was observed when compared to SIL-WT AMs (**Figure 7G**), suggesting an important role of Axl-mediated efferocytosis and inflammation control during silicosis. These findings suggest that the increased severity of silicosis in mice lacking Axl is most likely due to an accumulation of necrotic cells that were not cleared by AMs via the Axl receptor, contributing to an amplified inflammatory response and lung damage. Overall, our findings indicate that despite sharing several cognate characteristics with TAM receptor family members, Axl and MerTk play functionally distinct roles in the regulation of the lung microenvironment and are functionally distinct receptors that control lung function during homeostasis and inflammatory lung diseases, respectively.

## Discussion

TAM receptors have emerged as key players in immunoregulation because, in addition to efferocytosis, they limit TLR activation and cytokine production in innate immune cells (10, 22). Axl and MerTk receptors are differentially expressed in several tissues and on immune cells (14, 16) under tolerogenic and inflammatory settings (10, 13, 14, 23).

Our study characterizes the function of Axl and MerTk receptors *in vivo* in homeostatic and inflammatory settings in the airways and lung parenchyma. We showed that both Axl and MerTk receptors are constitutively expressed by lung cells and AMs, but MerTk had an important role in downregulating AM and neutrophil recruitment, the inflammatory AM phenotype, and the maintenance of high levels of TGF-β and IL-10 in the lungs during homeostasis. On the other hand, Axl deficiency impaired the regulation of silica-induced inflammation, which resulted in an increase in inflammatory cells, pro-fibrotic TGF-β and CXCL1, as well as apoptotic and necrotic cell debris, and a decrease in the number of non-inflammatory AMs. Finally, we demonstrated that Axl mRNA is increased in both MerTk^-/-^ and WT AMs, emphasizing its importance in efferocytosis and inflammatory regulation. This study provides evidences that MerTk and Axl are specialized to orchestrate apoptotic cell clearance in different settings, playing constitutive and significant roles in lung immunological homeostasis and resolution of silicosis, respectively, and may have significant implications for the development of emerging therapeutic approaches using different TAM agonists.

Understanding the impact of the microenvironment on Axl and MerTk expression is crucial for comprehending the dynamic changes that may occur during homeostatic versus inflammatory or infectious settings. Moreover, it will contribute to the development of new therapeutic strategies to modulate efferocytosis, particularly in tissues colonized by commensals and with constant cell turnover and apoptosis, such as the lungs. In the present work we show that during the steady state, lung cells express high levels of Axl, MerTk, and Gas6, but negligible levels of Tyro3, as expected. AMs are the main contributors to the resident phagocytic cell pool which mediates the rapid and efficient removal of the cellular ‘corpses’ during lung homeostasis (3, 7). Due to the relevant role of AMs in efferocytosis and controlling inflammation in innate immune cells, we confirmed the expression of both Axl and MerTk receptors in these cells (14). Interestingly, we found that Axl and MerTk receptors had a significant cell-type-specific expression profile, with higher levels of Axl expressed preferentially by the mucosal macrophages, AMs and cMPs. In agreement with previous studies demonstrating the induction of Axl during inflammation (14, 23), we found that Axl expression was even higher in cMPs than in AMs, which is correlated with the expected intestinal inflammatory tone due to the higher microbiota density and diversity present in the colon compared to the lungs (24–26). In addition, while being evenly distributed throughout all macrophage populations studied, MerTk expression was highest on AMs, indicating its relevance in lung homeostasis. Indeed, MerTk-deficient mice showed higher percentages and absolute numbers of AMs and neutrophils in their BALFs compared to WT or Axl-deficient mice, indicating an increase in lung inflammatory tone induced by deficiencies in efferocytosis mediated by MerTk on AMs. The higher numbers of neutrophils found in the MerTk-deficient BALFs could result from either increased neutrophil recruitment or an accumulation of dead (apoptotic and necrotic) neutrophils. However, the quantification of apoptotic and necrotic neutrophils in the BALFs during homeostasis has not been done, which is a limitation of our study.

Efferocytosis is primarily characterized by its anti-inflammatory or tolerogenic properties, which result in an increased production of regulatory molecules such as transforming growth factor-β (TGF-β), interleukin (IL)-10 among others (27). Our results also demonstrate that MerTk is important in maintaining TGF-β and IL-10 levels in the BALFs during steady state and then contributing to the maintenance of lung homeostasis (28). Although the airways represent a specific niche in the lungs, we found that MerTk has the same relevant role in parenchyma homeostasis by controlling inflammatory cell infiltration and avoiding tissue damage, most likely by downregulating CXCL1 and CXCL2 chemokines and TNF-α and IL-6 production, respectively. In addition to the production and release of regulatory molecules, TAM receptor-mediated efferocytosis dampens inflammation via a negative feedback involving the activation of suppressor of cytokine signaling 1 (SOCS1) and SOCS3, which inhibit the TLRs and cytokine receptor signaling pathways (9, 10, 22). This process is likely to be particularly important to the tolerance of microbiota and innocuous inhaled antigens in sites colonized and continually challenged by antigens, such as the lung mucosa. Indeed, the absence of MerTk signaling increased the frequency of M1-like MHCII^+^ AMs but no differences in the frequency of M2-like CD206^+^ AMs compared to Axl-deficient or WT mice were observed. The current findings suggest that in addition to efferocytosis (16), MerTk may be able to control the induction of inflammatory AMs phenotype and function even under microbiota-derived MAMPs stimulation, avoiding unnecessary inflammation and tissue damage in a steady state setting. Our findings are consistent with previous studies that found that MerTk signaling reduces M1 polarization in *in vitro*-stimulated macrophages (9, 29). However, to the best of our knowledge, the current work is the first demonstrating the role of MerTk in downregulating M1 population *in vivo* in a steady state setting.

Defective efferocytosis and, consequently, a failure to resolve inflammation has been linked to the development/worsening of a variety of lung chronic inflammatory disorders (30, 31). Moreover, many studies have demonstrated the relevance of TAM family members in the control of inflammation, as well as the use of TAM receptor agonists in the treatment of mouse models of inflammatory diseases (32). Previous studies have shown that Axl receptor activation attenuates ischemia-reperfusion-induced acute lung inflammation and injury (33) as well as asthma (31). In agreement, we found that mice lacking Axl are more susceptible to silicosis than MerTk^-/-^ or WT mice. Indeed, the SIL-Axl group had a 40% decrease in survival rate as well as reduced pulmonary function as indicated by increased airway resistance, which can also be caused by necrotic cell accumulation in the lungs. These functional changes were followed by lung parenchyma damage with more inflammatory cell infiltrates, collagen fiber deposition, and increased lung permeability in SIL-Axl. Furthermore, even in our acute silicosis model, we found that the SIL-Axl group had significantly higher levels of the pro-fibrotic cytokine TGF-β in their BALFs, indicating that Axl may play a crucial role in the modulation of the fibrotic response. Others, on the other hand, have demonstrated that Axl and/or Gas6 activity leads to the stimulation of pulmonary fibroblasts and TGF-β production in individuals with idiopathic pulmonary fibrosis (34).

AMs are maintained throughout the lifespan by self-renovation, and previous studies have demonstrated that during inflammation or infection, monocyte-derived AMs are recruited to work with resident AMs in the lungs (35). Here, we observed an increased number of AMs in SIL-Axl BALFs compared to SIL-MerTk or SIL-WT groups. In agreement, we had a higher percentage of AMs in Axl-SIL compared to WT-SIL, although all SIL groups exhibited a lower percentage of AMs than their PBS counterparts. The reduction in the frequency of AMs during silicosis is likely attributed to the elevated levels of neutrophils present in the BALFs. Furthermore, while it is still unknown whether resident AMs are plastic and change their phenotype during the early stages of silicosis or if they are derived from circulating monocytes (36), the current data show that the number of non-inflammatory M2-like CD206^+^MHCII^-^ AMs from Axl-SIL was dramatically reduced whereas the number of M1-like MHCII^+^CD206^-^ AMs was elevated, which likely contributes to chronic inflammation. Our findings are supported by a previous study showing that Axl signaling inhibition increases the number of M1 macrophages in the invasive pulmonary aspergillosis (IPA) model (37). On the other hand, another study found a decrease in M1-associated inflammatory factor production by tumor-associated macrophages in the MDA-MB-231 xenograft breast cancer model (38). Together, these findings suggest that Axl signaling can trigger or suppress the M1 phenotype in a variety of disease settings (39). Neutrophils also play an important role in the early stages of respiratory disorders, including silicosis (40). The role of Axl in the recruitment of neutrophils and production of inflammatory cytokines and chemokines has already been described during Influenza infection (14). In line with this, we found increased neutrophil recruitment as well as levels of the neutrophil-recruiting chemokine CXCL1 after silica instillation in SIL-Axl BALFs compared to SIL-WT groups.

Aside from the massive induction of apoptosis, the accumulation of apoptotic cells in the lungs during silicosis has been linked to defects in efferocytosis, which eventually leads to fibrotic disorders and the development of autoimmune diseases such as systemic sclerosis and systemic lupus erythematosus (41, 42). In addition, a very elegant study showed that exposure to silica may contribute to the impairment of the efferocytosis capacity of mouse and human macrophages (43).

Although many receptors are involved in efferocytosis by AMs, we found more apoptotic, late apoptotic, and necrotic AMs and neutrophils in Axl but not in MerTk-deficient or WT mice, contrasting with previous report that demonstrated that MerTk attenuated lung inflammation caused by chronic silica inhalation (44). These differences may be due to of two different disease time points (chronic versus acute). Finally, we found that MerTk^-/-^ AMs had significantly higher Axl expression than WT AMs, suggesting that the Axl receptor is both essential for clearing apoptotic cells and controlling inflammation during silicosis, in agreement with what was found in asthma (31) and influenza virus infection (14).

This study provides strong evidence that MerTk and Axl are specialized to orchestrate apoptotic cell clearance in different settings, where they play constitutive and significant roles in lung immunological homeostasis and silicosis resolution, respectively. While MerTk regulates inflammation in healthy tissues that are constantly exposed to microbiota, antigens, and cellular renewal, and in which billions of apoptotic cells are generated and cleared on a regular basis, Axl regulates inflammation and helps to restore homeostasis during silicosis. These findings may have significant implications for the understanding of different settings of pulmonary and systemic inflammatory disorders as well as for the development of emerging therapeutic approaches using TAM agonists.

## Data availability statement

The raw data supporting the conclusions of this article will be made available by the authors, without undue reservation.

## Ethics statement

The animal study was approved by Ethics Committee for Use of Animals of the Universidade Federal do Rio de Janeiro, under protocol No. 041/17. Committee/institutional review board: Prof. Marcel Frajblat - Instituto de Biofísica Carlos Chagas Filho, Universidade Federal do Rio de Janeiro; Prof. Mariana Boechat de Abreu - Instituto de Ciências Biomédicas, Universidade Federal do Rio de Janeiro; Prof Fernanda de Mello e Souza Valente Gubert – Instituto de Ciências Biomédicas, Universidade Federal do Rio de Janeiro. The study was conducted in accordance with the local legislation and institutional requirements.

## Author contributions

KG-P: Investigation, Formal Analysis, Writing – original draft, Writing – review & editing. ML: Methodology, Writing – review & editing. AC: Methodology, Writing – review & editing. EM: Methodology, Writing – review & editing. LR: Methodology, Writing – review & editing. AdC: Methodology, Writing – review & editing. TR: Methodology, Writing – review & editing. JR: Methodology, Writing – review & editing. TF: Methodology, Writing – review & editing. YJ-S: Methodology, Writing – review & editing. PS: Methodology, Writing – review & editing. EC-E: Methodology, Writing – review & editing. US: Methodology, Writing – review & editing. JH: Methodology, Writing – review & editing. AC-d-S: Methodology, Writing – review & editing. MFL: Resources, Writing – review & editing. HM-G: Resources, Writing – review & editing. BLK: Resources, Writing – review & editing. AF: Conceptualization, Formal Analysis, Funding acquisition, Project administration, Supervision, Writing – original draft, Writing – review & editing.
