# Supplementary material for: Differential regulation of lung homeostasis and silicosis by the TAM receptors MerTk and Axl
Source: Front Immunol. 2024 May 7;15:1380628. doi: 10.3389/fimmu.2024.1380628 (PMC11106457; doi:10.3389/fimmu.2024.1380628)
Supplement: Supplementary file 1 [file DataSheet_1.docx]

**Supplementary Methods**

**Histological Analysis.** Left superior lobes were perfused with 5 mL of sterile PBS, gently inflated with 1 mL of 10% buffered formalin, and embedded in paraffin. Five-micron tissue sections were stained with Hematoxylin and Eosin (H&E) or Picrosirius Red. For the quantification of lung parenchyma inflammatory areas, digital images were generated by Panoramic MIDI (3DHistech, Hungary) slide scanner and extracted using Panoramic Viewer Software (3D Histech, v.1.15.4) on a scale of 1:4. Single lung images were uploaded to ImagePro (Media Cybernetics, v.10.9), and the parenchyma was submitted to automated analysis to detect and quantify the total lung left superior lobe area, which was turned into a Region of Interest (ROI). Using the Smart Segmentation tool (Machine Learning), the software detected high-density areas (cell inflammatory infiltrates) and quantified the total area in each lobe. The total percentage of space occupied by cell inflammatory infiltrates was calculated as the equation: total cell inflammatory infiltrate area x 100/total lung area.

**Bronchoalveolar lavage and isolation of lung parenchyma cells.** Mice were anesthetized with an intraperitoneal (i.p.) injection of pentobarbital sodium (2.0 mg/kg) and euthanized by exsanguination by severing the inferior vena cava. Lavage of the upper airways was performed eight times with 1 mL of sterile PBS each. Cell-free supernatants from the first bronchoalveolar lavage fluid (BALF) containing airways cells were stored at -80ºC for further analyses. Alternatively, lungs were perfused with 5 mL of sterile PBS, removed from the thoracic cavity, and incubated in 5 mL of collagenase VIII (100 units/mL) (Sigma-Aldrich) in Petri dishes for 1 hour under agitation at 37ºC in a 5% CO_2_ humid atmosphere. Lung tissue was homogenized by mechanical dissociation, and cell suspensions were passed through a 70 μm strainer and centrifuged at 1,500 rpm for 5 min. Single-cell suspensions from lung parenchyma (lung cells) or from BALF (airway cells) were counted and used for mRNA profiling or stained for flow cytometry in a FACSCanto or BD LSRFortessa (BD) and data were analyzed using FlowJo software (Tree Star Inc.) after gating out dead cells and non-singlets.

**Isolation of macrophages from the colon and peritoneal cavity and differentiation of macrophages from bone marrow precursors.** Colonic macrophage isolation (cMPs) was prepared as described previously^77^. Briefly, entire colons from 30-50 WT mice were collected and washed with calcium and magnesium-free Hanks’ balanced salt solution (HBSS) (Gibco, Grand Island, NY). The tissues were opened and incubated with HBSS containing 0.015% dithiothreitol (DTT; Sigma-Aldrich) for 15 minutes, followed by extensive washing in cold HBSS containing 5% heat-inactivated fetal bovine serum (FBS) (GemCell, West Sacramento, CA, USA) to remove epithelial cells and mucus. Colon pieces were gently minced and digested in a digestion buffer for 60 minutes at 37º C. The samples were filtered, spin down, and resuspended in 1.077 g/cm^3^ Optiprep (Axis-Shield, Dundee, Scotland). After centrifugation at 2,000 rpm for 15 minutes at room temperature, the viable cells from the layer surface were collected and cMPs (MHCII^hi^F4/80^hi^CD64^+^) were sorted using FACSAria (BD). Resident pMPs were obtained by washing the peritoneal cavity of mice with cold RPMI 1640 (Gibco) and plated for 2 hours at 37º C in a 5% CO_2_. After incubation and washing step to remove nonadherent cells, adherent pMPs were collected. Bone marrow-derived macrophages (BMDMs) were generated by flushing femurs and tibias with PBS, and precursor cells were cultured in complete RPMI 1640 media (Gibco) supplemented with 30% L929 cell-conditioned medium as the source of macrophage colony-stimulating factor. After 7 days in culture, mature BMDMs were harvested using CellStripper (CellGro, Pittsburgh, PA).

**RNA isolation and real-time reverse transcriptase–PCR.** Lung tissues were homogenized with Lysing Matrix D (MP Biomedicals, CA, USA) using a Precellys homogenizer. For real-time reverse transcriptase–PCR analysis of gene expression, AMs, cMPs, BMDMs, pMPs and lung total cells were collected, and total RNA was isolated using an RNeasy Mini Kit (Qiagen, Valencia, CA) following the manufacturer’s instructions. cDNA was synthetized using qScript cDNA Supermix (Quanta Biosciences, Beverly, MA) and real-time PCR analysis was performed on the 7900HT Sequence Detection System using FAM-labeled gene-specific probes and primers (all from Applied Biosystems, MA, USA). The level of target gene expression was calculated as 2^−ΔCt^ where ΔCt=Ct_target_−Ct_GAPDH_, with glyceraldehyde 3-phosphate dehydrogenase (GAPDH) as endogenous control and Ct indicating threshold cycle.

**Protein and cytokines quantification.** Total protein concentration was measured in the first BALF fifteen days post-silica or PBS instillations by using the Micro BCA Protein Assay Kit (Thermo Scientific, MA, USA) following the manufacturer’s protocol. Briefly, cell-free BALF supernatant was distributed to a 96-well plate and the protein concentration was assessed by a BSA standard (200 - 0.5 μg/mL) following incubation at 37ºC with working reagent substrate. Plates were immediately read by spectrophotometer (SpectraMax M5, Molecular Devices, San Jose, CA, USA) at 562 nm. BALFs were used for the measurement of cytokines concentrations by conventional double-sandwich enzyme-linked immunosorbent assay (ELISA) kits from eBioscience (active TGF-β), R&D (Mineapolis, MN) (IL-10) or Peprotech (Cranbury, NJ) (CXCL1), respectively, according to the manufacturer’s instructions. Plates were immediately read by spectrophotometer (SpectraMax M5, Molecular Devices, San Jose, CA, USA) at 450 nm.
